# Myricetin Inhibits Osteosarcoma Cell Viability and Modulates EMT-Related Genes Associated with the *SNAI1/MMP-9* Axis

**DOI:** 10.3390/ph19030499

**Published:** 2026-03-18

**Authors:** Isabela Santos, Hélio M. T. Albuquerque, Marta Teixeira Pinto, Nuno Mendes, José Miguel P. Ferreira de Oliveira, Eduarda Fernandes

**Affiliations:** 1 Laboratório Associado para a Química Verde (LAQV), Rede de Química e Tecnologia (REQUIMTE), Faculty of Pharmacy, University of Porto, Rua de Jorge Viterbo Ferreira 228, 4050-313 Porto, Portugal; up202112015@up.pt; 2 Laboratório Associado para a Química Verde (LAQV), Rede de Química e Tecnologia (REQUIMTE), Department of Chemistry, Campus Universitario de Santiago, University of Aveiro, 3810-193 Aveiro, Portugal; helio.albuquerque@ua.pt; 3 i3S—Institute of Investigation and Innovation in Health, 4200-135 Porto, Portugal; mtpinto@i3s.up.pt (M.T.P.); nmendes@i3s.up.pt (N.M.); 4 Ipatimup—Instituto de Patologia e Imunologia Molecular da Universidade do Porto, 4200-135 Porto, Portugal

**Keywords:** bone cancer, flavonoids, EMT, chick chorioallantoic membrane

## Abstract

**Background/Objectives:** Osteosarcoma treatment options remain limited due to tumor metastasis and the toxicity of conventional chemotherapy, warranting new therapeutic strategies. A well-founded strategy is the use of flavonoids, a class of phytochemicals possessing pharmaceutical properties that contribute to anticancer effects, including antioxidant and anti-inflammatory properties. This study aimed to evaluate the anticancer potential of flavonoids in osteosarcoma and investigate their interaction with doxorubicin. **Methods:** In this study, five flavonoids were screened for cytotoxicity and selectivity across four osteosarcoma cell lines and healthy fibroblasts (MRC-5). The interaction between myricetin and doxorubicin was assessed using a fixed-ratio combination approach. Cell migration and invasion were evaluated using cell exclusion/wound healing and 2D co-culture assays. EMT-related gene expressions were assessed by RT-qPCR. Antitumor activity was evaluated in vivo using a chick chorioallantoic membrane (CAM) xenograft model. **Results:** Myricetin emerged as the most selective compound, exhibiting cytotoxicity against osteosarcoma cells while sparing MRC-5 fibroblasts. Notably, myricetin synergized with doxorubicin (ratio 69:1), enhancing its cytotoxicity and significantly reducing osteosarcoma cell migration in vitro. Myricetin downregulated *SNAI1* and *MMP9*, suggesting modulation of epithelial–mesenchymal transition (EMT)-related pathways. Complementarily, in the CAM xenograft model, myricetin reduced xenograft tumor size, confirming its anticancer activity in vivo. **Conclusions:** Collectively, these findings emphasize the anticancer potential of myricetin in osteosarcoma through inhibition of the *SNAI1/MMP-9* signaling axis.

## 1. Introduction

Primary bone cancers originate from bone and cartilage cells. Among the different types, osteosarcoma is the most common primary tumor occurring in fast-growing bones (mainly the femur and tibia) [1]. Osteosarcoma has a bimodal distribution, with incidence peaks at 18 and 60 years of age, occurring more frequently in males [2]. Regarding its treatment, a combination of methotrexate, doxorubicin, and cisplatin, also referred to as the MAP regimen, is used as the primary standard of care since the late 1980s. Therapeutic progress has stagnated, and overall survival has reached a plateau [3,4]. The clinical management of osteosarcoma faces two challenges: the early onset of lung metastases, which reduce overall survival to 10–20%, and the severe toxicity associated with high-dose chemotherapy [5]. Consequently, there is an urgent need for adjuvant therapies enhancing chemotherapeutic efficacy while targeting metastasis.

As in several other types of cancer, metastases represent a major challenge for therapeutic progress in osteosarcoma [6]. The plasticity and mobility acquired by cancer cells, which enable their migration, result from phenotypic and morphological alterations associated with epithelial-to-mesenchymal transitions (EMT). EMT is induced by tumor microenvironment stimuli, including growth factors, inflammatory cytokines, and extracellular remodeling (changes in density and rigidity) [7,8]. In parallel, transcription factors such as SNAI1/2, TWIST1/2, and ZEB1/2 are upregulated to repress epithelial markers such as E-cadherin and induce mesenchymal markers including N-cadherin and vimentin [9]. This phenotypic switch induces cancer cell plasticity and promotes migration and invasion. A hallmark of this aggressive phenotype is the upregulation of matrix metalloproteinases (MMPs), particularly MMP-2 and MMP-9, which degrade the extracellular matrix (ECM) [10,11]. Additionally, metastatic cells show increased chemoresistance, thereby reducing MAP effectiveness [5,12]. The efficacy of the MAP regimen is also constrained by dose-limiting toxicities. The incorporation of chemotherapeutic agents such as doxorubicin and cisplatin significantly improves therapeutic response; however, their systemic administration is associated with cumulative cardiotoxicity and nephrotoxicity, respectively [4,13,14]. Clinically, these adverse effects are managed through cumulative dose thresholds, cardiac monitoring, liposomal formulations, and dose adjustments, although these measures do not fully eliminate the risk, underscoring the need for new adjuvant therapies to improve outcomes [13,14].

Flavonoids are naturally occurring polyphenolic compounds abundant in the human diet that have gained attention for their antioxidant and anticancer properties [15,16,17,18,19]. Despite limitations in aqueous solubility and bioavailability, ongoing structural optimization and formulation strategies aim to overcome these pharmacokinetic constraints [20,21]. Notably, research evidence indicates that flavonoid anticancer activity extends beyond apoptosis induction, cell cycle disruption, and proliferation inhibition, also encompassing modulation and remodeling of the extracellular matrix (ECM) [22]. As previously demonstrated, treatment of osteosarcoma cells with robinetin modulates EMT through mesenchymal-related transcription factors such as SNAI1/2, TWIST, and ZEB, as well as by downregulation of N-cadherin expression over E-cadherin [23,24]. Flavonoids can also modulate the expression of MMP such as MMP-2 and MMP-9 and restore ECM homeostasis [25]. Flavonoids can act as adjuvant agents that enhance chemosensitivity, synergizing with doxorubicin to reduce its effective dose while maintaining anticancer efficacy [6,26]. Moreover, flavonoids have been reported to exhibit low toxicity toward healthy cells and tissues and exert protective effects that mitigate chemotherapy-induced side effects [27]. The versatility of these compounds underscores their therapeutic value for treatment of osteosarcoma.

This study focused on myricetin, selected from an initial screening of flavonoids for its potential to selectively target osteosarcoma cells while sparing healthy cells. Myricetin was further investigated for its ability to synergize with doxorubicin, modulate EMT-related factors, and reduce metastatic behavior in osteosarcoma models. To this end, myricetin was tested across four osteosarcoma cell lines and one non-malignant fibroblast cell line. We provide the first evidence of the therapeutic potential of myricetin in osteosarcoma since: (1) it acts synergistically with doxorubicin, inhibiting osteosarcoma migration and potentially enabling reduction in chemotherapeutic dosage; (2) it suppresses EMT-related genes, including *SNAI1* and *MMP-9*; and (3) it inhibits tumor growth in a concentration-dependent manner in an osteosarcoma xenograft model in vivo. Altogether, these results emphasize the anticancer potential of myricetin by inhibiting osteosarcoma cell viability, decreasing tumorigenesis, and modulating EMT-related genes through suppression of the *SNAI1*/*MMP-9* signaling axis.

## 2. Results

### 2.1. Cytotoxicity Screening in Osteosarcoma Cells

**Figure 1:**
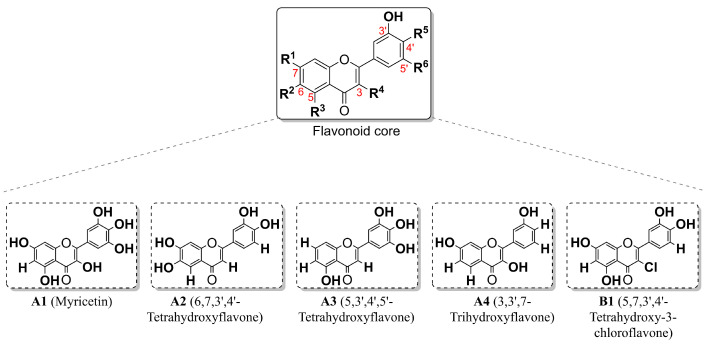
Flavonoids used in this study.

MG-63, Saos-2, HOS, and 143B osteosarcoma cell lines were incubated with five flavonoids for 48 h (Figure 1).

The WST-8 assay was conducted to measure cell viability, while the SRB assay was conducted to measure cell growth. Then, the concentration of the selected flavonoids that reduced cell viability or cell growth by 50% (half-maximal inhibitory concentrations, IC_50_) was determined and is presented in Table 1.

**Figure 2:**
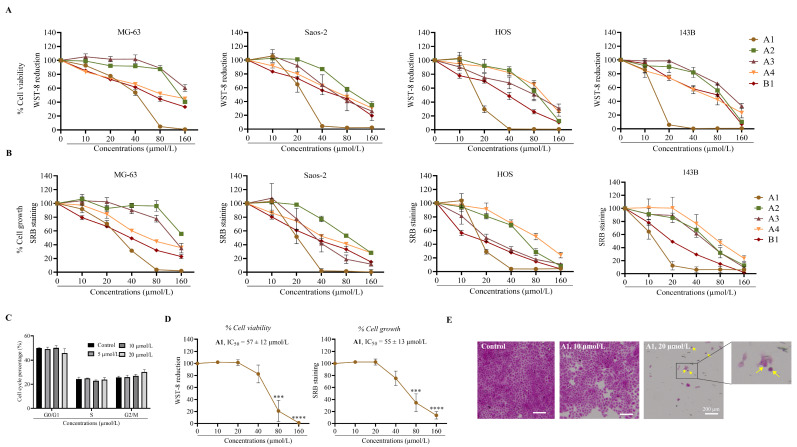
**Myricetin (compound A1) demonstrated the highest cytotoxicity against osteosarcoma cells and was selective to osteosarcoma versus MRC-5 fibroblasts.** Cell viability (**A**) was measured by WST-8 assay and cell growth (**B**) by SRB assay. Osteosarcoma cell viability and growth curves were obtained after exposure to flavonoids **A1** to **B1** at increasing concentrations (0–160 µmol/L) for 48 h. (**C**) Cell cycle analysis of 143B after treatment with myricetin; after 48 h of treatment, 143B cells were stained with propidium iodide (PI) to assess DNA content and analyzed by flow cytometry. The resulting histograms represent the distribution of the cells in different phases of the cell cycle. (**D**) Viability and growth curves of MRC-5 cells exposed to myricetin (**A1**, 0–160 µmol/L) for 48 h. (**E**) Representative brightfield images of 143B cells following treatment with myricetin (48 h exposure), fixation and SRB staining; yellow arrows indicate cellular blebs and cells undergoing cytoplasmic loss. The data are expressed as the mean ± SEM (*n* ≥ 3). Statistically significant differences (asterisks) are related to 0.1% DMSO control; *** *p* < 0.001; and **** *p* < 0.0001 (one-way ANOVA).

The cytotoxicity profiles are presented for cell viability (Figure 2A) or cell growth (Figure 2B) in the osteosarcoma cell lines.

Overall, cell viability and growth inhibition were concentration-dependent, as shown in Figure 2A,B and evidenced by their cytotoxicity profiles. Amongst the cell models used, 143B cells were the most sensitive to flavonoids **A1** to **B1**, highlighted by their lowest IC_50_ (48 h) value, followed by HOS and Saos-2. MG-63 cells were the least sensitive (Table 1).

The four osteosarcoma cell lines used in this study show morphological and phenotypic differences, allowing for a more robust assessment of compound effects in osteosarcoma: both MG-63 and Saos-2 exhibited osteoblastic phenotypes, with MG-63 less differentiated and highly proliferative, and Saos-2 more differentiated. On the other hand, HOS and 143B are described as the most aggressive osteosarcoma cells showing high capacity to metastasize and invade. Despite being derived from HOS cells, the introduction of a KRAS mutation confers a highly tumorigenic phenotype to 143B cells [27].

The use of structurally distinct flavonoids across different osteosarcoma cell lines enhances the robustness of the study, since variations in both cellular phenotypes and flavonoid chemical structures contribute to experimental variability. Based on the results from Figure 2A and Table 1, cell growth was generally more susceptible to flavonoid treatment rather than cell viability. Nevertheless, compound **A1** inhibited both cell growth and viability on osteosarcoma cells, with the strongest effects observed in HOS and 143B cells, marked by IC_50_ < 20 µmol/L (48 h, Table 1). Furthermore, compound **B1** reduced cell growth in HOS cells, IC_50_ < 20 µmol/L (48 h, Table 1). In contrast, compound **A3** exhibited greater viability inhibitory effects only in Saos-2 cells. Compounds **A2** and **A4** exhibited low toxicity towards osteosarcoma cells with IC_50_ values > 50 µmol/L (48 h, Table 1). Moreover, structure-related compounds allowed the identification of which substituents are more susceptible to inducing toxicity towards osteosarcoma cells: replacing hydrogen (compound **A2**, Figure 1 and Table 1) with chlorine (compound **B1**, Figure 1 and Table 1) in C-3 position increased the overall toxicity across all osteosarcoma cells for both cell viability and cell growth (Table 1); replacing the hydroxy groups at C-3 and C-7 (Figure 1, **A1**) by hydrogens (Figure 1, **A3**) sharply decreased cell viability and growth in all osteosarcoma cells. Overall, the hydroxy or chlorine groups in C-3 seem to enhance osteosarcoma toxicity.

Among the tested flavonoids, **A1** was the most effective against all the osteosarcoma cell lines tested in this study, which is highlighted by the lower IC_50_ values (48 h, Table 1). The cytotoxic effect was more pronounced in 143B (lowest IC_50_, 48 h, Table 1), followed by HOS, Saos-2, and MG-63. This first screen enabled the identification of myricetin as the most active compound against osteosarcoma; therefore, it was the compound chosen for the subsequent assays. In addition, due to the invasive and aggressive phenotype of 143B cells, this cell model was used in the subsequent assays. In addition to the cytotoxicity screening, we performed a cell cycle analysis on 143B cells. After 48 h of incubation with **A1** (myricetin), the results showed no significant difference between the control and treated groups (Figure 2C). The cellular blebbing, cytoplasmic loss, and increased cell debris observed with myricetin treatment (Figure 2E) together with the absence of significant alterations in the cell cycle (Figure 2C) suggest cytotoxic rather than cytostatic effects.

One of our goals, given the systemic toxicity of doxorubicin and cisplatin, was to identify a flavonoid that selectively inhibits osteosarcoma viability while minimizing toxicity towards a healthy cell model. We chose human lung fibroblasts as the healthy cell model to test the most active flavonoid, compound **A1**, since in metastatic osteosarcoma, lungs are the primary organ affected [28]. Myricetin exhibited low toxicity to MRC-5 cells, as demonstrated by the cytotoxicity profile (Figure 2D) and an IC_50_ of 57 ± 12 µmol/L (48 h), nearly 5 times higher than that obtained for the 143B cell line.

### 2.2. Myricetin and Doxorubicin Synergistically Decreased Osteosarcoma Viability

**Figure 3:**
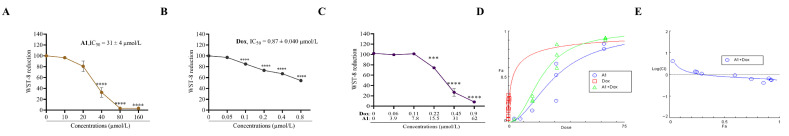
**Myricetin synergizes with doxorubicin potentiating the inhibition of osteosarcoma cell viability.** 143B osteosarcoma cells were treated with myricetin (**A1**; 0–160 µmol/L), doxorubicin (Dox; 0–0.8 µmol/L), or their combinations for 24 h. Cell viability analysis (WST-8 assay) and corresponding IC_50_ value for (**A**) myricetin, (**B**) doxorubicin, and (**C**) myricetin dox combinations. (**D**) Fraction affected (Fa) inhibition curve and (**E**) logarithmic combination index (CI) versus Fa for combinations of myricetin with doxorubicin. **A1**: myricetin; Dox: doxorubicin. The data are presented as the mean ± SEM (*n* ≥ 3). Significant differences (asterisks) are shown relative to 0.1% DMSO control (concentration 0); *** *p* < 0.001 and **** *p* < 0.0001 (one-way ANOVA).

To evaluate the potential of myricetin as adjuvant therapy, its interactions with doxorubicin were evaluated in 143B cells. Using the WST-8 assay, and after 24 h incubation, myricetin showed an IC_50_ value of 31 µmol/L and doxorubicin an IC_50_ value of 0.87 µmol/L, with the corresponding cytotoxicity profiles shown in Figure 3A,B. The concentration range of myricetin and doxorubicin combinations was set based on the obtained IC_50_ values following 24 h exposure. Compound interactions were analyzed using CompuSyn software, establishing a constant molar ratio mode. CompuSyn provides information in the form of an inhibition curve of the fraction affected (Fa) (Figure 3C) and logarithmic combination index (LogCI) versus Fa (Figure 3D).

CompuSyn analysis indicates that the combination index (CI) is < 1, showing that combination of myricetin and doxorubicin with a defined ratio (69:1) synergized, inhibiting 143B viability. Synergy became more pronounced at higher effect levels (CI = 0.61 at Fa 0.9; Table 2).

### 2.3. Synergistic Effect of Myricetin and Doxorubicin on the Inhibition of 143B Cell Migration

**Figure 4:**
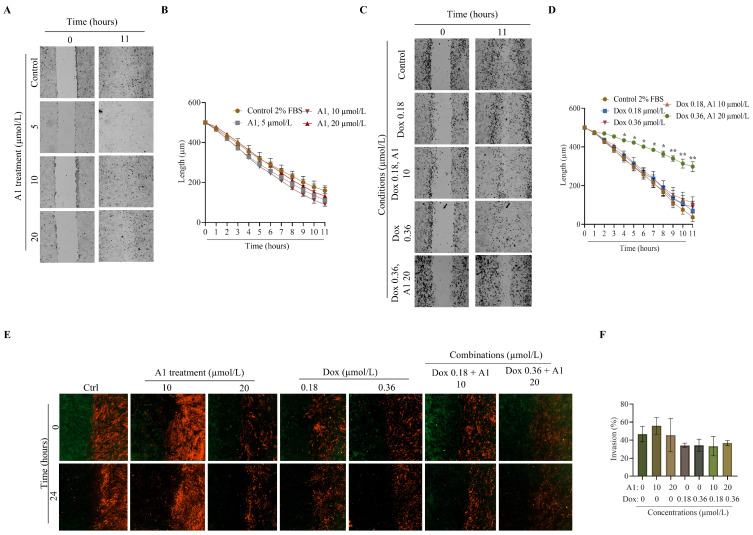
**Synergistic action of Myricetin and Doxorubicin efficiently inhibits osteosarcoma cell migration.** (**A**) Representative images of 143B cells following treatment with myricetin. (**B**) Quantification of the average width of 143B cell migration following myricetin treatment alone. (**C**) Representative brightfield images of 143B cells gap after exposure to myricetin/doxorubicin combinations. (**D**) Quantification of the average width of 143B following treatment with myricetin/doxorubicin combinations. (**E**) Representative images of 143B cell invasion at 0 and 24 h, following exposure to myricetin or myricetin/doxorubicin combinations. MRC-5 lung cells were stained with Cell TrackerTM Red CMTPX dye and 143B osteosarcoma cells with Cell TrackerTM Green CMFDA dye. (**F**) Quantification of the invading rate of 143B cells (green). **A1**: myricetin; Dox: doxorubicin. Data are presented as mean ± SEM (*n* ≥ 3). Significant differences (asterisks) are shown relative to solvent control; * *p* < 0.05 and ** *p* < 0.01 (two-way ANOVA).

Migration and invasion contribute to the spread of osteosarcoma, correlating with poor patient outcomes. We therefore investigated the effects of myricetin, doxorubicin, and their combinations on 143B cell migration and 2D invasion, using a cell exclusion zone assay. Cell migration was monitored for 11 h, and 2D invasion for 24 h. A cell-free area (gap) was created, separating 143B cells (migration) (Figure 4A,C) or 143B and MRC-5 cells (invasion) (Figure 4D); then, cells were incubated with different doses of myricetin, doxorubicin, or myricetin/doxorubicin combinations.

Although treatment with myricetin did not significantly reduce gap closure compared to solvent control (Figure 4A,B), a trend toward decreased migration was observed in the treated groups versus control, particularly at later time points (Figure 4B). Doxorubicin alone, at either concentration, did not significantly reduce gap closure. However, the combination of doxorubicin 0.36 µmol/L with myricetin 20 µmol/L significantly inhibited cell migration from 4 h of incubation onwards, while the lower combination (doxorubicin 0.18 µmol/L + myricetin 10 µmol/L) showed no significant effect (Figure 4C,D). The 11 h point was selected to capture the full extent of migration, as the cell-free area became closed after approximately 11 h, as confirmed in Supplementary Figure S1, which displays time-framed representative images of gap closure acquisitions of control with 2% FBS and combination of doxorubicin (0.36 µmol/L) with myricetin (20 µmol/L).

Additionally, we explored the effects of myricetin, doxorubicin, or myricetin/doxorubicin combinations in 143B cell invasion, in 2D co-culture with MRC-5 fibroblasts (Figure 4E,F). Under the conditions of the assay, neither myricetin nor doxorubicin alone, nor their combinations, significantly prevent the invasion of 143B cells into the MRC-5 occupied area (Figure 4F), suggesting that the compounds exert a more pronounced effect on migration that on 2D invasion (matrix-free system) under these conditions.

### 2.4. Myricetin Downregulated EMT-Related Transcription Factors in Osteosarcoma Cells

Epithelial–mesenchymal transition (EMT) is a coordinated cellular process in which transcription factors such as Snail (*SNAI1*) and Zeb1 (*ZEB1*) regulate the epithelial-to-mesenchymal shift. Additionally, metalloproteinases contribute to extracellular matrix (ECM) degradation, enabling cancer cells to migrate and invade surrounding tissues [12].

**Figure 5:**
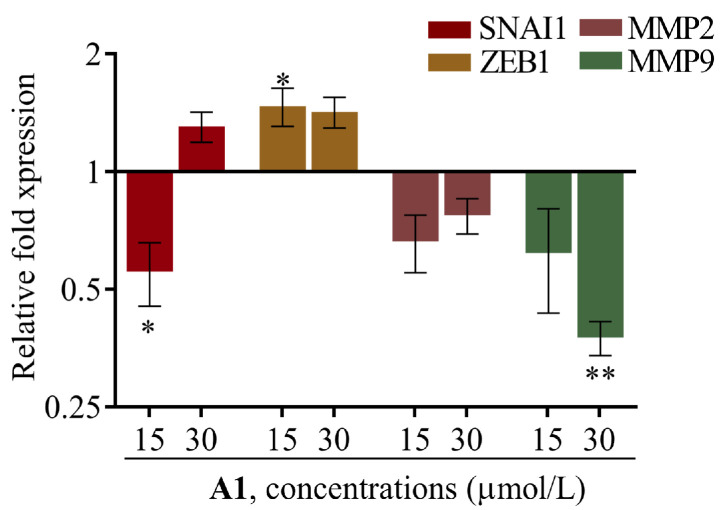
**Myricetin downregulates *SNAI1* and *MMP-9* in osteosarcoma cells.** RT-qPCR was performed to evaluate the gene expression levels of EMT-related transcription factors (*SNAI1*, *ZEB1*) and matrix metalloproteinases (*MMP2* and *MMP9*) in the 143B metastatic cell line. After treatment with myricetin for 24 h, gene expression was normalized to a reference gene and calculated relative to the control group. The data are expressed as mean ± SEM (n = 3). Significant differences (asterisks) are shown relative to 0.1% DMSO control; * *p* < 0.05 and ** *p* < 0.01 (one-way ANOVA).

The expression of *SNAI1*, *ZEB1*, and *MMP-2/9* genes was therefore evaluated by RT-qPCR in 143B cells treated with myricetin at 15 and 30 μmol/L, for 24 h (Figure 5).

Gene expression analysis demonstrated that treatment with myricetin significantly increased the expression of *ZEB1* at the lower concentration tested—15 μmol/L, but not at the higher concentration of 30 μmol/L. Additionally, myricetin treatment at 15 μmol/L significantly reduced *SNAI1* expression. Regarding the expression of metalloproteinases, myricetin treatment did not significantly affect the expression of *MMP-2*. However, it significantly reduced the expression of *MMP-9* at 30 μmol/L. Overall, these results suggest that myricetin modulates EMT-related gene expression by downregulating *SNAI1* transcription factor and *MMP-9* metalloproteinase in 143B cells.

### 2.5. Myricetin Exhibited Antitumor Activity by Reducing Tumor Growth in CAM Xenografts

The in vivo antitumorigenic effect of myricetin was assessed using a chick embryo model xenografted with 143B cells. The chick chorioallantoic membrane (CAM) assay is an in vivo model that aligns with the 3Rs policy (Replace, Reduce, Refine) and has been used in studies related to drug efficacy and tumor growth. In the early stages of chick embryo development, the embryos are immunodeficient, facilitating the acceptance of cancer cells regardless of their origin [28].

**Figure 6:**
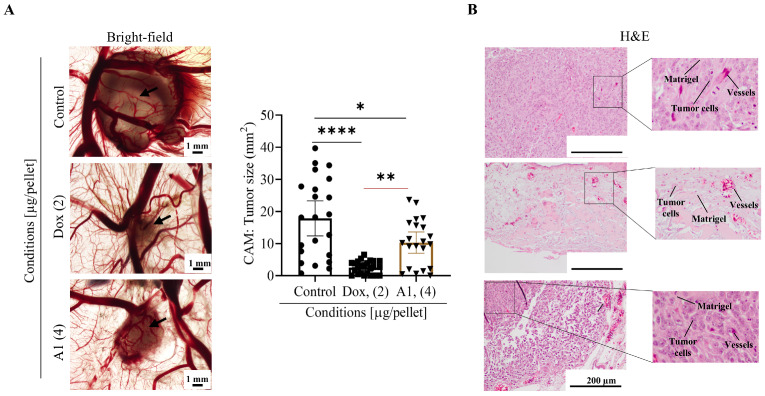
**Myricetin suppresses tumor growth in the CAM assay.** 143B cells in Matrigel were mixed with saline vehicle, doxorubicin (2 µg), or myricetin (4 µg) and xenografted onto the E9 chick chorioallantoic membrane (CAM), then allowed to grow for 7 days. At E16, the xenografted CAMs were excised, and tumor areas were measured. (**A**) Representative brightfield images of 143B xenograft tumors and graphical analysis of the tumor size of each group. Dispersion analysis with 95% confidence intervals is depicted in the image (*n* ≥ 3, eggs per replica ≥ 5). Significant differences are represented by asterisks, * *p* < 0.05, ** *p* < 0.01, and **** *p* < 0.0001 (one-way ANOVA). **A1** (4): myricetin (4 µg/pellet); Dox (2): doxorubicin (2 µg/pellet). The black arrows indicate the inoculation site. (**B**) Hematoxylin and eosin (H&E) staining of CAM xenografts.

In this study, the chemotherapeutic agent doxorubicin was used as a reference, as its effects on CAM tumors are already described, and it is the most commonly used in cancer treatment [24,29]. Furthermore, a saline solution containing 1% DMSO was used as a solvent control (vehicle), allowing for a comparison between treated and untreated tumors. After nine days of chick embryo development (E9), the eggs were incubated with 143B cells resuspended in Matrigel and test compound in solvent (proportion of 1:1). After seven days, at E16, the embryos were euthanized, the CAMs were excised and tumor areas were measured. CAM xenografts were further processed for histologic analysis (Figure 6).

According to the histological presentation, in comparison to the vehicle control group, treatment with myricetin or doxorubicin resulted in smaller tumor areas. Statistical analysis supports the histological observations by demonstrating that treatment with myricetin or doxorubicin, in comparison to control conditions, significantly reduced the size of the CAM tumors (*p* = 0.0100 and *p* < 0.0001, respectively) (Figure 6A). Moreover, H&E tumor sections corroborate the brightfield observations: an increase in cell-free Matrigel areas (Figure 6B, H&E) indicates loss of cell viability, tumor shrinkage, and less invasive tumors. Overall, these observations from CAM model support and validate the in vivo antitumorigenic role displayed of myricetin.

## 3. Discussion

Osteosarcoma is the most common aggressive primary bone tumor and therapeutic advances have stagnated, resulting in a plateau in overall survival. The current standard of care relies on the application of MAP regimen chemotherapy; however, it fails to adequately address (i) pulmonary metastases, that sharply decrease the overall survival to 10–20%; (ii) chemoresistance, and (iii) systemic toxicity associated with conventional chemotherapy [3,5]. These limitations hinder treatment options and underscore the need for novel therapeutic strategies [30]. Flavonoids are a class of naturally occurring polyphenolic compounds widely recognized for their antioxidant properties and a broad spectrum of biomedical activities, including anticancer activity [17]. However, their clinical translation is hindered by poor solubility, low bioavailability, and rapid metabolism, challenges that nanotechnology has been helping to mitigate through advanced delivery systems. Myricetin specifically has a reported oral bioavailability in preclinical pharmacokinetic studies of approximately 9.6–9.7% due to poor absorption, with a time to maximum blood concentrations (T_max_) of approximately 6.4 h, reflecting its limited absorption and solubility [31]. Despite these limitations, flavonoids have emerged as promising anticancer candidates, acting through multiple mechanisms, including inhibition of cancer cell proliferation, induction of apoptosis, cell cycle arrest, and suppression of invasion and migration [15,16]. Additionally, they alter the expression of transcription factors (e.g., SNAI1, ZEB1) and effectors (e.g., MMP-2/9), modulating signaling pathways involved in epithelial–mesenchymal transition (EMT) [23,24].

To address the current limitations of osteosarcoma treatment, we screened a panel of flavonoids to identify compounds with high cytotoxicity and selectivity toward osteosarcoma cells while sparing healthy cell models. SAR analysis revealed that the pyrogallol moiety in the B-ring, combined with hydroxy groups at positions 3, 5, and 7, significantly contributed to cytotoxicity in osteosarcoma, while replacement of the C-3 hydroxy group with chlorine enhanced selectivity toward HOS cells. From the initial screening, myricetin (**A1**) emerged as the most selective compound toward osteosarcoma cells, most prominently against 143B cells, a highly metastatic KRAS mutant that recapitulates aggressive disease in vitro. Although myricetin has been studied in other cancer types, it remains largely unexplored in osteosarcoma [32,33]. Notably, the IC_50_ values (48 h) observed here are comparable to or lower than those reported for other types of cancer: Shih et al. reported that myricetin at concentrations ≥ 30 µmol/L for 48 h significantly affected A549 lung cancer cell viability [34]; Ye et al. reported an IC_50_ of 47.6 µmol/L in PC3 cells, suggesting that osteosarcoma cells, including the 143B cell line, may be particularly sensitive to myricetin [35].

Despite its dose-limiting toxicity and emergence of chemoresistance, doxorubicin remains the most widely used chemotherapeutic agent in osteosarcoma therapy [36]. Combination strategies have emerged as a promising approach to overcome these limitations while maintaining therapeutic efficacy [37]. In this context, myricetin demonstrated synergistic interactions with doxorubicin, reducing the effective dose of doxorubicin while preserving anticancer activity. These findings are consistent with reports showing similar synergy between doxorubicin and other flavonoids, such as 3′,4′-dihydroxyflavonol [38]. Regarding cell motility, myricetin alone did not significantly affect migration or invasion, which may reflect the sub-cytotoxic concentrations used in these assays. In contrast, the combination of myricetin with doxorubicin significantly inhibited 143B cell migration but not 2D invasion. Similar observations for cancer cell migration were reported by Yunita et al., where hesperidin in combination with doxorubicin significantly decreased the migration of AT1 breast cancer cells [39].

Beyond cell viability, myricetin’s modulation of EMT-related regulators such as SNAI1 and ZEB1, or effectors such as MMP-2/9, may contribute to the anti-migratory effects observed when combined with doxorubicin. While no significant downregulation was observed for *ZEB1* or *MMP2*, myricetin downregulated *SNAI1* at 15 µmol/L and *MMP9* at 30 µmol/L, with a trend to increase from 0 and 15 µmol/L to 30 µmol/L, supporting a role for myricetin in limiting metastatic potential. In our study, however, *MMP-9* downregulation did not translate into significant inhibition in the 2D invasion assay, a discrepancy that warrants consideration. This may partly reflect inherent limitations of the invasion model, particularly the absence of a defined physical ECM barrier. Two major hypotheses arise: first, MRC-5 fibroblasts may not have produced a sufficiently dense ECM to create a stromal barrier, limiting the functional relevance of MMP activity in this context. Second, as described by Wolf et al., the mesenchymal–amoeboid transition (MAT) can enable tumor cells to invade through physical displacement independently of proteolytic ECM degradation, a mechanism that may predominate in environments where proteolytic degradation is not strictly necessary (e.g., 2D co-cultures or loose matrices), potentially explaining why MMP-9 transcriptional downregulation did not translate into reduced invasion [30].

Previous studies have reported that myricetin modulates EMT by directly suppressing transcription factors such as *SNAI1* or downregulating effectors such as MMPs. Ci et al. demonstrated that treatment of MDA-Mb-231Br breast cancer cells with 5 or 10 µmol/L myricetin inhibited migration and invasion through dose-dependent suppression of MMP-2 and MMP-9 expression [17,32]. Similarly, Chen et al. showed that the structurally related flavonoid quercetin reduces *SNAI1* expression in triple-negative breast cancer cells via inhibition of the IGF1/IGF1R-Akt/ERK pathway, thereby attenuating EMT and metastatic potential [40]. Together with our findings, these results suggest that modulation of *SNAI1* and *MMP9* expression may represent a common mechanism underlying the anti-metastatic properties of flavonoids, supporting the role of myricetin as a potential EMT modulator in osteosarcoma.

These in vitro findings were further evaluated using a CAM xenograft model with 143B osteosarcoma cells. The CAM assay is a well-established in vivo platform commonly used for the preliminary evaluation of tumor growth and drug responses in a cost-effective and reproducible manner [24,29,41]. Notably, after treatment with myricetin, CAM tumors were significantly smaller when compared to vehicle control; however, doxorubicin exhibited stronger inhibitory effect, as expected for a standard chemotherapeutic agent.

While this study provides valuable mechanistic and translational insights, several limitations should be acknowledged. First, vitro analyses were primarily conducted in 143B cells, and validating the effects of myricetin in additional osteosarcoma cell lines would strengthen the generalizability of our findings. Second, mechanistic analyses were largely based on gene expression due to its sensitivity. Complementary assessment at the protein level, including post-translational modifications such as phosphorylation, would further clarify the mechanism of action and the potential involvement of TGF-β/MAPK signaling pathways. Third, the 2D invasion assay lacks a dense ECM barrier, limiting the ability to directly evaluate the functional role of MMP-9 in invasion. Finally, while the CAM xenograft offers advantages for early-stage in vivo studies, it does not fully capture the cellular heterogeneity and microenvironmental complexity of human osteosarcoma.

In conclusion, this study demonstrates that myricetin exhibits selective cytotoxicity toward 143B osteosarcoma cells, synergizes with doxorubicin to reduce the required therapeutic dose, and modulates EMT-related gene expression by downregulating *SNAI1* and *MMP9*. These effects were validated in a CAM xenograft model, supporting the antitumor potential of myricetin in vivo. Altogether, these findings present myricetin as a promising candidate for osteosarcoma therapy, warranting further investigation into molecular targets, protein-level effects, and efficacy in more complex preclinical models.

## 4. Materials and Methods

### 4.1. Cell Culture Media and Reagents

Dulbecco’s modified Eagle’s medium (DMEM) (catalog no. 31053044), fetal bovine serum (FBS) (catalog no. 10500056), L-glutamine (catalog no. 25030024), penicillin–streptomycin (catalog no. 15140122) and trypsin-EDTA (0.25% Trypsin, 1 mM EDTA) (catalog no. 25200072) were purchased from Thermo Fisher Scientific (Carlsbad, CA, USA). The following reagents were obtained from Sigma-Aldrich, Inc. (St. Louis, MO, USA): dimethyl sulfoxide (DMSO, catalog no. 472301), Dulbecco’s phosphate-buffered saline (DPBS, catalog no. D1408), sulforhodamine B (SRB, catalog no. S1402), trichloroacetic acid (TCA, catalog no. T6399), CCK-8 kit (containing WST-8 reagent, catalog no. 96992). Sodium chloride (NaCl, catalog no. AC424290250) was obtained from Fisher Scientific (Hampton, NH, USA). The assayed compounds myricetin (**A1**, 021127S); 6,7,3′,4′-tetrahydroxyflavone (**A2**, 22-393); and 3,3′,7-trihydroxyflavone (**A4**, 22-327) were purchased from Indofine Chemical Company, Inc. (Hillsborough, NJ, USA). Compounds 5,3′,4′,5′-tetrahydroxyflavone (**A3**) and 5,7,3′,4′-tetrahydroxy-3-chloroflavone (**B1**) were obtained by synthesis according to previous publications [42,43]. The cell tracer CytoTrace Red TM CMTPX dye (red, catalog no. AAT-22015) was obtained from AAT Bioquest (Pleasanton, CA, USA) and Cell TrackerTM Green CMFDA dye (green, catalog no. HY-126561-1mg) from MedChemExpress (Monmouth Junction, NJ, USA).

### 4.2. Cell Culture Reagents and Exposure Conditions

The four human osteosarcoma cell lines (MG-63, Saos-2, HOS, and 143B) and fetal human lung fibroblast (MRC-5) were purchased from the European Collection of Authenticated Cell Cultures (ECACC, Salisbury, UK) in September 2020 and authenticated by Short-Tandem Repeat (STR) profiling. Upon arrival, cells were cryopreserved from each of 3 subsequent passages. After thawing, cells were subcultured for 4 months. Routine procedures included mycoplasma testing and cell maintenance in complete medium supplemented with 10% FBS, 2 mM L-glutamine, 100 U/mL penicillin, and 100 µg/mL streptomycin. 143B cell line maintenance included the addition of bromodeoxyuridine (at a final concentration of 15 mg/L) to the complete medium. Cells were maintained at 37 °C in a humidified atmosphere containing 5% CO_2_. Subculturing involved cellular detachment with 0.25% trypsin-1 mM EDTA. Flavonoid compound solutions were kept at −20 °C and were prepared to obtain a concentration of 100 mmol/L in DMSO. The DMSO final concentration was kept at 0.1% in complete DMEM.

### 4.3. Cytotoxicity and Cell Growth Quantification

The cytotoxicity assays were performed in 96-well plates. After adhesion of osteosarcoma cells (5 × 10^3^ cells/well) or MRC-5 lung fibroblasts (1 × 10^4^ cells/well), flavonoids (10, 20, 40, 80 and 160 μmol/L) or doxorubicin (0, 0.05, 0.1, 0.2, 0.4 and 0.8 μmol/L) solutions were prepared and added to the cells and incubated for 48 h. Additionally, the most active compound and doxorubicin were also evaluated in a 24 h cytotoxicity assay. Cytotoxicity was measured with WST-8 [2-(2-methoxy-4-nitrophenyl)-3-(4-nitrophenyl)-5-(2,4-disulfophenyl)-2H-tetrazolium, monosodium salt] assay using the Cell Counting Kit-8 (Sigma-Aldrich, St. Louis, MO, USA). WST-8 is an extracellular tetrazolium dye that in viable cells is rapidly reduced by NAD(P)H to produce a formazan product that can be quantified by reading the absorbance at 450 nm. The absorbance of formazan is proportional to the number of viable cells [44]. After incubation with flavonoids, culture medium was removed and a solution containing 1:10 (*v*/*v*) CCK-8 reagent was added to the cells for 2 h at 37 °C. After incubation, absorbance was measured at 450 nm in a Synergy HT Multi-mode Microplate Reader (BioTek Instruments, Winooski, VT, USA).

Cell growth was assessed by the SRB assay and performed in sequence with the cell viability assay. After the cell viability assay, cells were fixed for 1 h with 10% (*w*/*v*) trichloroacetic acid (TCA) at 4 °C. The microplate was then washed with water and dried. Following, the SRB solution (0.05% *w*/*v* in 1% acetic acid) was added and incubated for 30 min. SRB reagent is a pink amino xanthene dye with two sulfonic groups that under slightly acidic conditions binds to basic amino acid residues. Under basic conditions, the protein-bound SRB is dissolved and the absorbance at 510 nm is directly proportional to the cell mass [45]. The excess dye was removed by washing the stained cells four times with 1% acetic acid (*v*/*v*). After drying, 10 mM Tris-base (unbuffered, pH ~10.5) was added, followed by shaking for 10 min. The absorbance was measured at 510 nm in a Synergy HT Multi-mode Microplate Reader (BioTek Instruments).

### 4.4. Combination Assay with Doxorubicin

In a 96-well plate, a density of 5 × 10^3^ cells/well of 143B was plated. After cellular adhesion, myricetin (0, 3.9, 7.8, 15.5, 31 and 62 µmol/L), doxorubicin (0, 0.06, 0.11, 0.22, 0.45 and 0.9 µmol/L) and myricetin/doxorubicin combinations were prepared with a constant ratio and added to each well for 24 h. Combination experiments were performed using a constant-ratio design based on the relative IC_50_ values of myricetin and doxorubicin, following the Chou–Talalay method implemented in CompuSyn. Concentrations were selected to include multiple points both above and below the IC_50_ values of the individual compounds. Cell viability was measured using Cell Counting Kit-8. Briefly, cell medium was removed, WST-8 solution in a 1:10 dilution was added and cells were incubated for 2 h. Subsequently, the absorbance at 450 nm was measured in a Synergy HT Multi-mode Microplate Reader (BioTek Instruments, Winooski, VT, USA). The data obtained were processed using CompuSyn software (version 1.0) and assuming constant ratios.

### 4.5. Migration and Invasion Assays

Migration and invasion assays were performed in 24-well plates. Cell migration was assessed by a cell exclusion/wound-healing assay and invasion was assessed by a 2D invasion assay [46]. Cells were detached using trypsin for 4 min and pellets were collected after centrifugation for 4 min at 200× *g*. Cell culture inserts were used to ensure a well-defined and uniform gap. FBS concentration was maintained at 2%, which is the minimum percentage that ensures the viability of 143B and MRC-5 throughout the assay. Myricetin (5, 10, and 20 µmol/L), doxorubicin (0.18 and 0.36 µmol/L), and myricetin/doxorubicin combinations were prepared in DMEM supplemented with 2% FBS and 0.1% DMSO.

For the migration assay (wound-healing assay), 143B cells were resuspended to a final cellular density of 8 × 10^5^ cells/mL. Then, 70 µL of cell suspension was applied into each well of an ibidi Culture-Insert 2 Well system (Ibidi, Gräfelfing, Germany). After an overnight period to promote cellular adhesion to the plate, the cell culture insert was removed, and the cells were washed with warm DPBS. Stock solutions of myricetin, doxorubicin, and myricetin/doxorubicin combinations were prepared and added to the plate. Medium containing 2% FBS and 0.1% DMSO was used as solvent control. Gap closure was monitored hourly for 11 h using the Cytation Gen 5 equipment (BioTek Instruments, Winooski, VT, USA), maintaining cells at 37 °C with 5% CO_2_. The collected image data were processed using ImageJ software (version 1.8.0), and at least three independent assays were conducted for each condition. For each image, 9 sections were defined and measured.

For the 2D invasion assay, 143B and MRC-5 pellets were resuspended in serum-free medium containing 10 µmol/L green CMFDA (143B cells) and 5 µmol/L red CMTPX (MRC-5 cells), for 15 min. After dye incorporation, cellular suspensions were centrifuged at 200× *g* for 4 min and then resuspended in complete DMEM to achieve final cell densities of 8 × 10^5^ 143B cells/mL and 4 × 10^5^ MRC-5 cells/mL. Then, 75 µL of each cell suspension was transferred to ibidi Culture-Insert 2 Well system (Ibidi, Gräfelfing, Germany) on a 24-well plate. To promote cellular adhesion, the plate was left in the incubator, and after overnight incubation, the cell culture inserts were removed, and the cells were washed with warm DPBS. Subsequently, 1 mL of medium containing 2% FBS was added to each well and cells were incubated for 14 h to allow gap closure. Stock solutions of myricetin (10 and 20 µmol/L), doxorubicin (0.18 and 0.36 µmol/L), and myricetin/doxorubicin combinations were prepared and added to the corresponding well. Invading osteosarcoma cells were incubated at 37 °C in 5% CO_2_ in a Cytation Gen 5 equipment and photographed hourly for 24 h. The collected images were analyzed using QuPath 0.5.1 software, and at least three independent assays were performed [47].

### 4.6. RNA Extraction, cDNA Synthesis and qPCR

After treatment with myricetin, gene expression was analyzed by qPCR. Briefly, 75 × 10^5^ 143B cells were cultured in T25 flasks and placed in a CO_2_ incubator to promote cellular adhesion. The following day, the culture medium was exchanged for complete medium containing myricetin (15 and 30 µmol/L) or the solvent control (0.1% DMSO) and cells were incubated for 24 h. Thereafter, cells were harvested and lysates were obtained using NZYol reagent (NZYtech, Lisbon, Portugal), according to the manufacturer’s instructions. An equal volume of chloroform was added and mixed by hand for 15 s. The aqueous and the organic phases were separated using Phase Lock Gel Heavy tubes (5 PRIME Inc., Boulder, CO, USA), according to instructions, which includes a centrifugation at 12,000× *g*, 15 min, 4 °C. After centrifugation, the aqueous top phase containing the RNA was transferred to a 1.5 mL RNase-free tube. To each sample collected, 1 mL of isopropanol (−20 °C) was added, and the samples were left at −20 °C for 15 min followed by another cycle of centrifugation at 12,000× *g*, 10 min, 4 °C. The resultant supernatant was carefully removed, and the pellet was washed with 1 mL of RNase-free 75% ethanol. After resuspension, the samples were submitted to another centrifugation at 12,000× *g*, 5 min, 4 °C and after careful removal of the ethanolic solution, the resultant RNA pellets were left in RNase-free environment to allow the solvent to evaporate at room temperature. Finally, the dried RNA pellets were resuspended in RNA-free water.

Before cDNA synthesis, RNA quality and purity were evaluated by plotting the absorbance spectrum from 230 to 280 nm and by measuring the 260/280 nm absorbance ratio, respectively, using the Synergy HT Multi-mode Microplate Reader TAKE 3 (BioTek Instruments, Winooski, VT, USA). Then, total RNA was quantified using the Agilent BioTek Take3 Multi-Volume Plate (Agilent, Santa Clara, CA, USA). From 5 μg of total RNA, first-strand cDNA was synthesized using Maxima H minus First Strand cDNA synthesis kit (Thermo Scientific, Waltham, MA, USA), according to the kit’s instructions. Subsequently, Primer3 was used to design forward (F) and reverse (R) gene-specific primers: *SNAI1*: F: TAGGCCCTGGCTGCTACAA, R: GCCTGGCACTGGTACTTCTT; *ZEB1*: F: CTGCCCAGTTACCCACAATC, R: TGTCTTTCATCCTGATTTCCATT; *MMP-2*: F: AGGGCACATCCTATGACAGC, R: AGTTCCCACCAACAGTGGAC; *MMP-9*: F: GTGCTCTTCCCTGGAGACCT, R: TCGACTCTCCACGCATCTC. *TBP* (TATA-Box Binding Protein, reference gene) F: AGGATAAGAGAGCCACGAACC, R: GCTGGAAAACCCAACTTCTG.

All cDNA samples were prediluted 1:5 (*v*/*v*) in ultrapure water. Each qPCR reaction contained: NZYSpeedy qPCR Green Master Mix (2x) (NZYtech, Lisbon, Portugal); 150 nmol/L of each gene-specific primer; 1:4 (*v*/*v*) prediluted cDNA (final 1:20). After adding the components to a 96-well plate, the qPCR cycling program was conducted using a qTOWER3 G thermal cycler (Analytik Jena, Thuringia, Germany), and included: 2 min of denaturation at 95 °C followed by 60 cycles of 95 °C for 3 s, 58 °C for 5 s, and 62 °C for 20 s. The qPCR ended with a melting program of increasing temperature from 55 °C to 95 °C using a rate of 1 °C per cycle. The final melting program was used to validate PCR specificity.

For each condition and gene tested, three independent assays were conducted. After cDNA synthesis, each of the three independent samples was analyzed by qPCR in technical triplicates for each gene. Gene expression levels were normalized to the reference gene (TBP) and calculated relative to the solvent control. Gene expression was calculated according to the 2^−∆∆Ct^ method [48,49].

### 4.7. Chicken Embryo (CAM Assay) Xenograft for Tumorigenesis Assessment

Chicken embryo chorioallantoic membrane (CAM) assay model was used to evaluate tumor growth in vivo, as previously described [29]. According to the European Directive 2010/63/EU and Portuguese law, ethical approval is not required for the experiments using embryonic chicken. Eggs obtained from commercial sources were incubated horizontally at 37.5 °C in a humidified atmosphere and referred to embryonic day (E). At E3, a window was opened, and 1.5–2 mL of albumen was removed to promote the development of the CAM detached from the shell. Thereafter, transparent adhesive tape was used to seal the window, and the eggs returned to the incubator. Solutions of myricetin or doxorubicin were prepared in saline (0.9% NaCl) with 1% DMSO; 1 × 10^6^ 143B cells were resuspended in myricetin (4 µg) or doxorubicin (2 µg) in saline solution with a final mixture volume of 5 µL. Then, cells with compounds were mixed with 5 µL of cold Matrigel (Corning, NY, USA). The final volume of 10 µL was carefully pipetted on to the E9 CAMs, into a silicone ring, under sterile conditions. At E16, the CAMs were fixed using a solution containing 10% buffered formalin and excised from the embryos. The tumors were photographed ex ovo, and their area was determined using the Olympus cellSens Standard 1.14 program. Tissue sections were embedded in paraffin, and the slide sections were stained with hematoxylin–eosin. For each condition, at least 11–13 eggs were used, and at least three independent assays were performed. Tumor treatments and area measurements were randomized and assessed blindly.

### 4.8. Statistical Analysis

For each analysis and methodology, at least three independent experiments were performed to ensure reproducibility and statistical rigor. In vitro assays (WST-8, SRB, migration, invasion) were conducted using cells from different passage numbers and weeks, with at least two technical replicates per independent experiment. For the CAM xenograft assay, each independent experiment included 11–13 eggs (biological replicates), and tumor area was quantified individually for each egg. Data were analyzed using GraphPad Prism 9 software (San Diego, CA, USA). Half-maximal inhibitory concentration (IC_50_) values were calculated using a nonlinear dose–response inhibition model. Results are presented as the mean ± standard error of the mean (SEM). Comparisons between control and treated groups were performed using one-way analysis of variance (ANOVA), followed by Dunnett’s post hoc test.

For drug interaction analyses, fraction affected (Fa) values and logarithmic combination index (LogCI) versus Fa plots were generated using CompuSyn software. Additional graphs were produced using GraphPad Prism 9. Statistical significance was defined as * *p* < 0.05, ** *p* < 0.01, *** *p* < 0.001, and **** *p* < 0.0001.

## 5. Conclusions

This study presents several key strengths: (i) identification of myricetin as a selective flavonoid against osteosarcoma cells, while sparing healthy fibroblasts; (ii) demonstration of synergistic effects between myricetin and doxorubicin, with potential to enhance chemotherapy efficacy, including anti-migratory effects, while potentially reducing associated toxicity; (iii) evidence that myricetin suppresses EMT-related genes, including *SNAI1* and *MMP-9*; and (iv) validation of anti-tumor activity in vivo through reduced tumor size in a CAM xenograft model. Collectively, these findings underscore the therapeutic potential of myricetin in osteosarcoma and provide a preclinical framework for further translational investigation.

## Figures and Tables

**Table 1:** **Half-maximal inhibitory concentration values in osteosarcoma cell lines (48 h incubation).** Osteosarcoma cells (MG-63, Saos-2, HOS, and 143B) were exposed to flavonoids **A1** to **B1** for 48 h.

	WST-8 ^1^ Assay (IC_5O_^2^ ± SEM ^3^)				SRB ^4^ Assay (IC_5O_ ± SEM)			
	MG-63	Saos-2	HOS	143B	MG-63	Saos-2	HOS	143B
**A1** (Myricetin)	38 ± 2	23 ± 2	18 ± 1	12 ± 2	28 ± 2	21 ± 1	18 ± 1	10 ± 1
**A2** (6,7,3′,4′-Tetrahydroxyflavone)	142 ± 3	106 ± 6	83 ± 11	75 ± 14	168 ± 3	88 ± 4	52 ± 2	55 ± 7
**A3** (5,3′,4′,5′-Tetrahydroxyflavone)	178 ± 9	36 ± 8	87 ± 22	111 ± 4	128 ± 11	37 ± 9	22 ± 5	53 ± 4
**A4** (3,3′,7-Trihydroxyflavone)	106 ± 3	71 ± 1	101 ± 12	56 ± 5	74 ± 1	54 ± 4	78 ± 6	75 ± 10
**B1** (6,7,3′,4′-Tetrahydroxy-3-chloroflavone)	60 ± 1	52 ± 3	36 ± 5	54 ± 5	40 ± 4	35 ± 3	14 ± 2	21 ± 2

^1^ [2-(2-methoxy-4-nitrophenyl)-3-(4-nitrophenyl)-5-(2,4-disulfophenyl)-2H-tetrazolium, monosodium salt]; ^2^ Half-maximal inhibitory concentration; ^3^ Standard error of the mean; ^4^ Sulforhodamine B.

**Table 2:** **CompuSyn Analysis after treatment of 143B cells with** **myricetin–doxorubicin (69:1)**.

**CI**	0.98	0.87	0.8	0.74	0.70	0.66	0.61
**Fa**	0.30	0.40	0.50	0.60	0.70	0.80	0.90
**Total Dose (A1 + Dox)**	15	18	21	26	31	40	57

**A1**: myricetin; CI: combination index; Dox: doxorubicin; Fa: fraction affected; Total dose: sum of the concentrations.

## Data Availability

The original contributions presented in this study are included in the article. Further inquiries can be directed to the corresponding authors.

## Supplementary Materials

The following supporting information can be downloaded at: https://www.mdpi.com/article/10.3390/ph19030499/s1, Figure S1: Effects of myricetin and doxorubicin in osteosarcoma cell migration. Representative images were acquired at different time points (0 to 11 h) and delimitation of the cell-free area.

## Abbreviations

The following abbreviations are used in this manuscript:

CAM	Chick Chlorioallantoic Membrane
CI	Combination index
Dox	Doxorubicin
ECM	Extracellular Matrix
EMT	Epithelial to mesenchymal transitions
Fa	Fraction affected
