# Supplementary figures and images for: Myricetin Inhibits Osteosarcoma Cell Viability and Modulates EMT-Related Genes Associated with the SNAI1/MMP-9 Axis
Source: Pharmaceuticals (Basel). 2026 Mar 18;19(3):499. doi: 10.3390/ph19030499 (PMC13028739; doi:10.3390/ph19030499)

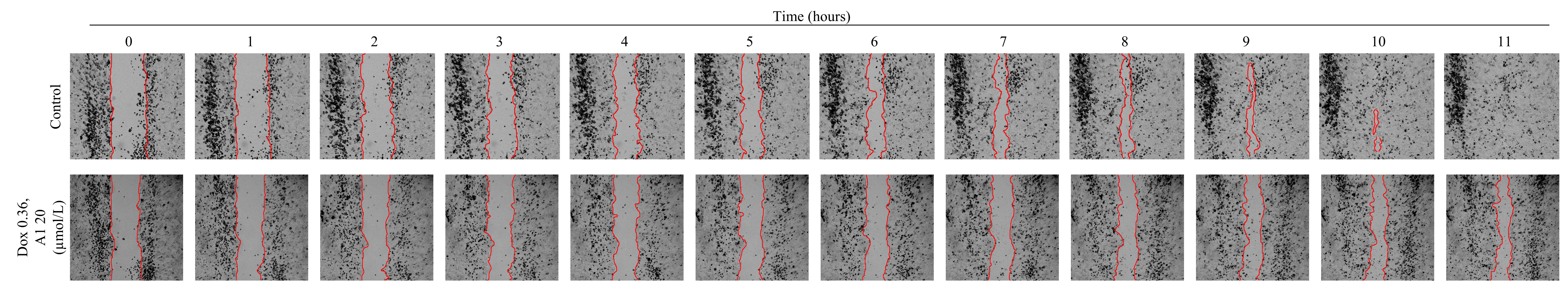

Supplement: Supplementary file 1 [file pharmaceuticals-19-00499-s001.zip › pharmaceuticals-4169147-supplementary.tif]
